# Diagnostic Value of White Blood Cell and C-Reactive Protein in Pediatric Appendicitis

**DOI:** 10.1155/2016/6508619

**Published:** 2016-05-04

**Authors:** Sevgi Buyukbese Sarsu, Fatma Sarac

**Affiliations:** ^1^Department of Pediatric Surgery, Cengiz Gokcek Obstetrics and Children's Hospital, Sehitkamil, 27560 Gaziantep, Turkey; ^2^Department of Pediatric Surgery, Haseki Training and Research Hospital, 34440 Istanbul, Turkey

## Abstract

*Background.* Acute appendicitis (AA) associated with acute phase reaction is the most prevalent disease which requires emergency surgery. Its delayed diagnosis and unnecessarily performed appendectomies lead to numerous complications. In our study, we aimed to detect the role of WBC and CRP in the exclusion of acute and complicated appendicitis and diagnostic accuracy in pediatric age group.* Methods.* Appendectomized patient groups were constructed based on the results of histological evaluation. The area under a receiver operating characteristic (ROC) curve (AUC) was performed to examine diagnostic accuracy.* Results.* When WBC and CRP were used in combination, based on cut-off values of ≥13.1 × 10^3^/*μ*L for WBC counts and ≥1.17 mg/dL for CRP level, diagnostic parameters were as follows: sensitivity, 98.7%; specificity, 71.3%; PPV, 50.6%; NPV, 99.5%; diagnostic accuracy, 77.6%; LR(+), 3.44; LR(−), 0.017. AUC values were 0.845 (95% CI 0.800–0.891) for WBC and 0.887 (95% CI 0.841–0.932) for CRP.* Conclusions.* For complicated appendicitis, CRP has the highest degree of diagnostic accuracy. The diagnosis of appendicitis should be made primarily based on clinical examination, and obviously more specific and systemic inflammatory markers are needed. Combined use of cut-off values of WBC (≥13100/*μ*L) and CRP (≥1.17 mg/L) yields a higher sensitivity and NPV for the diagnosis of complicated appendicitis.

## 1. Introduction

As a clinical entity, AA progresses with systemic inflammatory response, and it is the most frequent cause of acute abdomen [1]. Characteristic symptoms of appendicitis include vomiting (96%), fever (85%), and right lower quadrant abdominal pain (81%) [2]. Diagnosis of appendicitis is made primarily according to clinical manifestations. However, in children it has an atypical clinical presentation, and its symptoms resemble those of intussusception, gastroenteritis, pneumonia, and many other diseases [3]. Besides, classical symptoms detected in adults are not encountered in children. Since pediatric patients experience difficulties in cooperation, their abdominal examination is more challenging. In particular, in patients smaller than 4 years of age, the incidence of complicated appendicitis at admission is relatively higher [4]. Delay in diagnosis can result in serious complications as diffuse peritonitis, liver abscess, abdominal or retroperitoneal abscess, phlegmon, intestinal obstruction, bacteremia, sepsis, necrotizing fasciitis [5], and appendicovesical fistula [6, 7]. Compared with the adult population, incidence of appendiceal perforation caused by delayed diagnosis is more frequently observed in children [8]. As reported in many studies, unnecessarily performed appendectomies not only lead to formation of bridles but also can result in increased incidence of mortality [9].

Mostly luminal occlusion due to various etiologies and bacterial infections of the appendix are held responsible in the pathogenesis of this disease which is mostly seen in adolescents [10]. Progression of the inflammatory process increases intraluminal pressure leading to necrosis of the appendiceal wall. This condition has revived the use of inflammatory markers. The most widely used markers are WBC counts and CRP values. However, use of these parameters has yielded diverse and controversial results. Though CT, laparoscopy, and ultrasound have been used for diagnostic purposes, they could scarcely decrease the number of surgeries [11]. This condition has arisen the need to search for other inflammatory markers. However, none of the markers have demonstrated 100% diagnostic accuracy up to now [11]. In the decision-making process of diagnosis and surgery, clinical experience of the surgeon, anamnesis, and clinical symptoms are more significant than biochemical test results and imaging studies [12, 13].

The objective of this retrospective study is to determine NPV, PPV, and LR by using ROC curves estimated based on the most sensitive and specific cut-off values of WBC and CRP in their individual and combined uses in diagnosing or excluding acute and complicated appendicitis in pediatric patients.

## 2. Material and Methods 

This retrospective study was performed on successive 417 children with right lower abdominal quadrant pain and 126 healthy children who presented to our hospital between December 2014 and December 2015.

This study was performed following approval obtained from General Secretariat of Association of Public Hospitals. The ethics committee of Gaziantep University Faculty of Medicine approved the study (conclusion number 22.05.2014/26).

Laboratory tests were carried out on admission to hospital before administration of antibiotherapy.

In all patients, white blood cell counts were performed using automated hemocytometer (Beckman Coulter Inc., Brea, CA/USA) and CRP levels were measured using immunonephelometric methods (Beckman Coulter Inc., Brea, CA/USA). Normal values for WBC and CRP were accepted as 4.5–11 × 10^3^/*μ*L and <0.6 mg/dL, respectively.

### 2.1. Statistical Methods

For statistical analysis, Statistical Package for Social Sciences for Windows (SPSS) (SPSS Inc., version 15.0 software, Chicago, Illinois, USA) was used. For descriptive statistics, categorical variables were expressed as numbers, percentages, and numerical variables as means ± standard deviation (SD). For comparisons between two independent groups, Mann-Whitney *U* test was used. Comparison of categorical variables between groups was performed using* chi*-square test. For describing the diagnostic properties of WBCs and CRP levels, we used the area under ROC curve (AUC) and likelihood ratio (LR). The cut-off value was finally chosen to compare sensitivity, specificity, PPV, and NPV for each variable. All results were reported within 95% confidence intervals (95% CIs). *p* value of <0.05 was accepted as the level of statistical significance.

## 3. Results 

The study population consisted of 349 male and 194 female children aged between 6 and 17 years (median age, 11.09 years) who were divided into 5 groups as follows: Group 1 (126/543), healthy controls (they did not have right lower abdominal quadrant pain); Group 2 (100/543), children whose right lower abdominal quadrant pain originated from causes other than appendicitis but regressed during follow-up; Group 3 (39/543), children with histologically normal appendices; Group 4 (199/543), patients with AA; Group 5 (79/543), cases with complicated appendicitis. By definition, complicated appendicitis includes perforation of the appendix, empyema or abscess formation, and finally fecal peritonitis. Appendectomized patient groups were constructed based on the results of histological evaluation.

We demonstrated that the patients had right lower quadrant (RLQ) abdominal pain that originated from causes other than appendicitis with diagnostic tests (alone or in combination) for diagnosing appendicitis, clinical signs (e.g., psoas sign, obturator sign, Rovsing sign, and McBurney sign), clinical symptoms (e.g., fever, migrating pain, and guarding), laboratory tests (e.g., white blood cell count, C-reactive protein concentration, and left shift), and imaging tests (e.g., abdominal X-ray; US; CT with or without contrast administered orally, rectally, or intravenously). Finally, diagnostic laparoscopy was also used for the evaluation of patients with RLQ pain/suspected acute appendicitis. We used test combinations (as listed above) with clinical observation.

**Figure 1:**
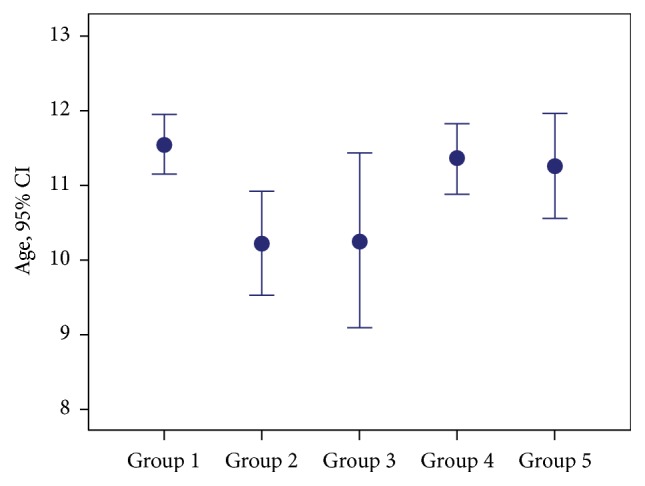
Distribution of groups according to age groups.

**Figure 2:**
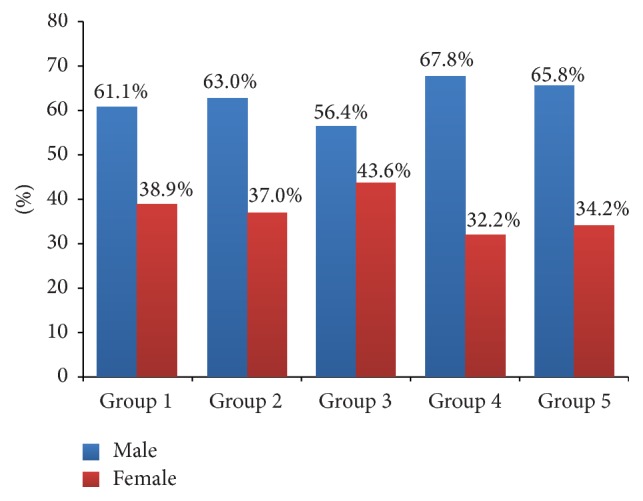
Distribution of groups based on the gender of the patients.

Mean age of Group 2 was lower relative to Groups 1 and 4 (*p* = 0.001, *p* = 0.004). Any intergroup difference was not detected between other groups. Male/female ratios of group did not differ statistically significantly (*p* = 0.583) (Table 1; Figures 1 and 2).

Mean WBC and CRP values were statistically significantly different between groups (for both *p* < 0.001).

**Figure 3:**
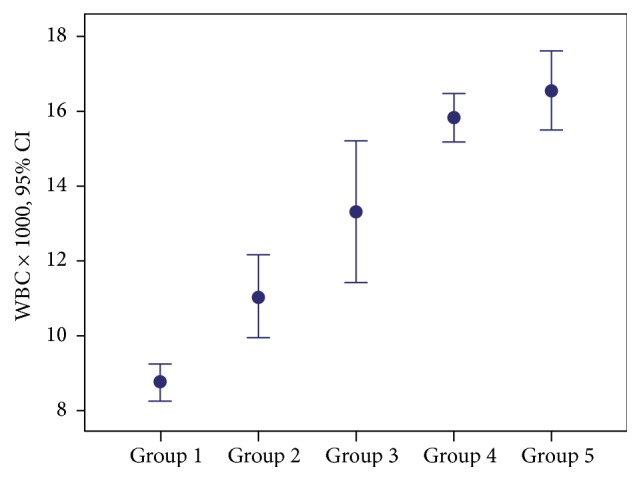
Distribution of groups based on mean WBC counts.

**Figure 4:**
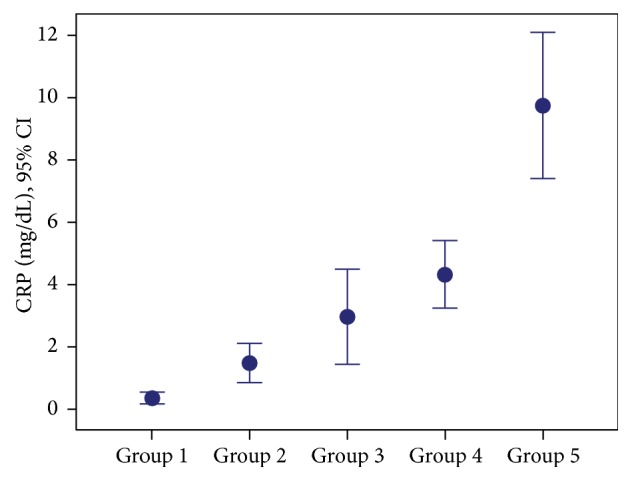
Distribution of groups based on mean CRP values.

Mean WBC counts and CRP values in Groups 4 and 5 were statistically significantly higher than those of Groups 1, 2, and 3 (for both *p* < 0.001) (Table 2; Figures 3 and 4).

Distribution of patients between groups based on normal or elevated WBC counts and CRP values are shown in Table 3.

In patients with AA, diagnostic parameters for white blood cell counts were as follows when cut-off value of ≥13.1 × 10^3^/*μ*L was taken into consideration: the highest degree of sensitivity, 73.4%; specificity, 80.0%, positive predictive value (PPV), 73.4%; negative predictive value (NPV), 80.0%; diagnostic accuracy, 77.2%.

Diagnostic parameters for CRP values were as follows when cut-off value of ≥0.6 mg/dL was taken into consideration: the highest degree of sensitivity, 70.9%; specificity, 68.7%; PPV, 62.9%; NPV, 75.8%; diagnostic accuracy, 69.6%.

In combined use, based on the cut-off values of WBC (≥13 × 10^3^/*μ*L) and CRP (≥0.6 mg/dL), diagnostic parameters were as follows: sensitivity, 95.5%; specificity, 60.8%; PPV, 64.6%; NPV, 94.7%; diagnostic accuracy, 75.7%; positive likelihood ratio [LR(+)], 2.43; negative likelihood ratio [LR(−)], 0.074.

**Figure 5:**
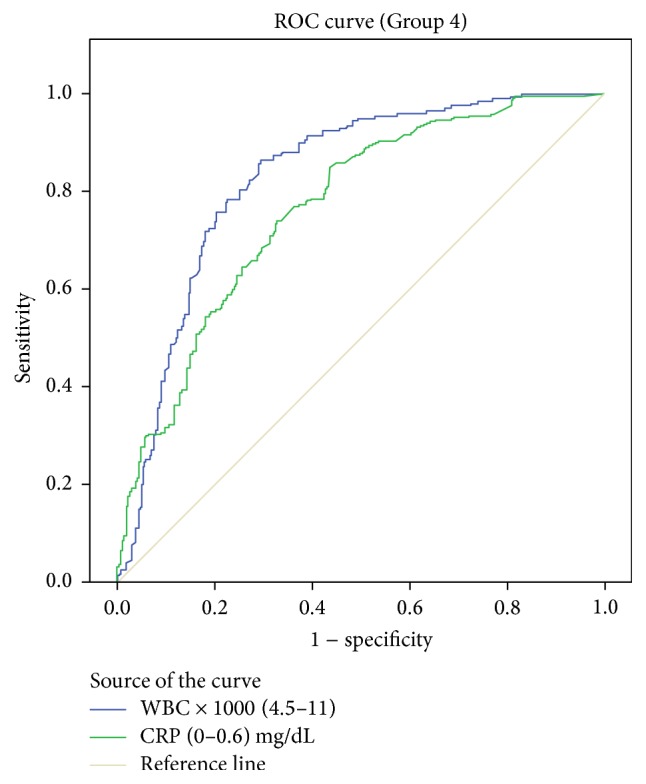
Receiver operating characteristic curves with corresponding area under the curve (AUC) for white blood cell (WBC) count and C-reactive protein (CRP) in predicting acute appendicitis.

AUC values were 0.828 [95% confidence interval (CI) 0.791–0.866] for WBC and 0.765 [95% CI 0.723–0.808] for CRP (Figure 5).

In patients with complicated appendicitis, when cut-off value for WBC counts was taken as ≥13.1 × 10^3^/*μ*L, diagnostic parameters were as follows: sensitivity, 78.5%; specificity, 80.0%; PPV, 53.9%; NPV, 92.6%; diagnostic accuracy, 79.7%.

When CRP cut-off value was taken as ≥1.17 mg/dL, diagnostic parameters were as follows: sensitivity, 86.1%; specificity, 81.9%; PPV, 58.6%; NPV, 95.2%; diagnostic accuracy, 77.7%.

When WBC and CRP were used in combination, based on cut-off values of ≥13.1 × 10^3^/*μ*L for WBC counts and ≥1.17 mg/dL for CRP level, diagnostic parameters were as follows: sensitivity, 98.7%; specificity, 71.3%; PPV, 50.6%; NPV, 99.5%; diagnostic accuracy, 77.6%; LR(+), 3.44; LR(−), 0.017.

**Figure 6:**
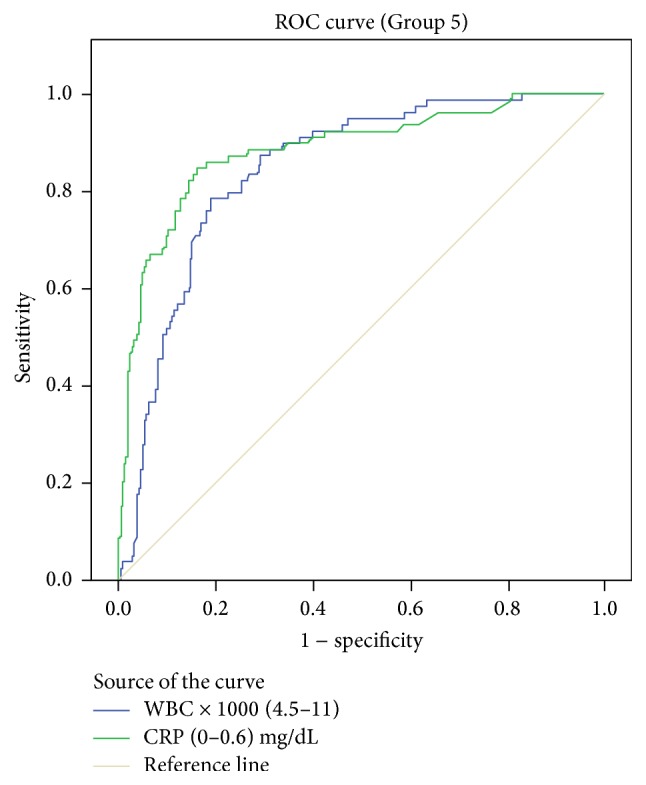
Receiver operating characteristic curves with corresponding area under the curve (AUC) for white blood cell (WBC) count and C-reactive protein (CRP) in predicting complicated appendicitis.

AUC values were 0.845 (95% CI 0.800–0.891) for WBC and 0.887 (95% CI 0.841–0.932) for CRP (Figure 6).

## 4. Discussion

Appendicitis is the most prevalently encountered entity of the pediatric age which requires urgent abdominal surgery [14]. Diagnosis is generally based on medical history, clinical evaluation, and physical examination. Nearly one-third of the cases progress with atypical clinical symptoms. Establishment of diagnosis is very difficult especially in the pediatric age group. Use of computed tomography and ultrasound could only decrease the incidence of negative appendectomy slightly. Since body fat is relatively less prominent in children, it is difficult to differentiate bowels from inflamed appendix. Therefore, in adults, CT has higher sensitivity and specificity (97 and 94%, resp.), while in children it has a relatively lower (50%) diagnostic accuracy. Besides, CT is more costly since it requires sedation of the patients beforehand. CT exposes the patients to radiation, and moreover sensitivity reaction can develop against the contrast agent used.

This phenomenon has revived the need for more effective use of inflammatory markers in the establishment of diagnosis. Recently, many novel inflammatory markers have been used to confirm the diagnosis of appendicitis; the most prevalently used laboratory markers for reinforcing the diagnosis are still white blood cell counts and C-reactive protein.

In AA, occlusion of the appendiceal lumen leads to impairment of blood flow and mucosal disruption. Afterwards, bacteria proliferate and leukocytic infiltration develops on this defective area. Migration of leukocytes to target tissues results in release of cytokines like CRP.

CRP, which is an acute phase protein synthetized from hepatocytes, was discovered in 1930 by Tillett and Francis. CRP synthesis increases within 4–6 hours after acute tissue injury or onset of the inflammation and doubles every 8 hours thereafter peaking at nearly 36–50 hours. Since its half-life is only 4–7 hours, its concentration rapidly drops. Therefore, in patients whose symptoms manifest within less than 12 hours, it has a relatively lower sensitivity.

It has been reported that, in more than 20% of pediatric patients with AA, WBC can be within normal limits [15]. In another study, the authors demonstrated that WBC counts are not adequately sensitive and specific in the discrimination between cases with or without appendicitis and also between acute and complicated appendicitis. However, it has been reported that CRP used solely or in combination with WBC is effective in the discrimination between acute and complicated appendicitis. Also in another study, only 7 of 100 successive patients with histopathologically confirmed AA WBC and CRP values were found to be within normal limits [15]. However, in our study, statistical significance of WBC and CRP values increased in parallel with the severity of the disease (for both, *p* < 0.001).

In many studies, increase in WBC has been reported as the earliest sign of appendiceal inflammation, while increase in CRP levels has been indicated in more advanced stages of appendicitis. In our study, among 32 cases with WBC within normal limits, 24 acute and 8 complicated appendicitis cases were diagnosed, and in 70 cases with normal CRP values 61 acute and 9 complicated appendicitis cases were detected. In a study group of 100 pediatric patients, diagnostic sensitivities of 0.60 and 0.86 were reported for increases in WBC and CRP, respectively. However, in our study among patients whose WBC and CRP levels were within normal limits, diagnoses of AA (*n* = 5/199; 2.51%) and complicated appendicitis (1/79; 1.26%) have been made in respective number of patients. Therefore, CRP had the highest diagnostic accuracy in complicated appendicitis (AUC: 0.887). As a consequence, CRP level should be routinely evaluated in patients with initial diagnosis of appendicitis [16, 17].

Contrary to descriptive and comparative statistical methods, ROC curve analysis allows evaluation of appropriateness of diagnostic parameters and diagnostic accuracy. LR(+) is described as the true-positive rate over the false-positive rate. It allows the physician to evaluate the likelihood that a case with a given test result (i.e., CRP level or elevated WBCs count) has that disorder.

Besides, based on cut-off values we estimated, we could obtain the values with utmost sensitivity and specificity.

In the literature, the sensitivity and specificity of WBC counts have been reported as 19%–88% and 53%–100%, respectively. However, in our study, corresponding rates were found to be 73.4 and 80.0%, respectively.

In AA, sensitivity and specificity of CRP have been reported as 48–75% and 57–82%, while in our study corresponding values were 70.9 and 68.7%, respectively.

We think that these variations in sensitivity and specificity arise from the time interval between the onset of the abdominal pain and evaluation of these markers.

In our study in patients with complicated appendicitis when WBC counts were used in combination with CRP, sensitivity and NPV increased to 98.7 and 99.5%, respectively, while specificity regressed to 71.3 percent. PPV and diagnostic accuracy were 50.6% and 77.6%, respectively. Compared with PPV, negative predictive values are more helpful in making an accurate diagnosis.

Our study population which also included patients without appendicitis but with right lower abdominal quadrant pain urged us to investigate negative predictive values of inflammatory markers. We detected higher sensitivity and NPV with combined use of CRP and WBC relative to their individual uses.

In various studies performed in pediatric patients, diagnostic markers as IL8, IL10, granulocyte, colony-stimulating factor, interferon *γ*, intercellular adhesion molecule-1, and matrix metalloproteinase-9 have not been useful in the differential diagnosis of abdominal pain. Still, in making a diagnosis of appendicitis, many novel markers as procalcitonin have been used whose diagnostic superiority over WBC and CRP has not been demonstrated so far [18, 19].

Equal diagnostic values of clinical examination and recurrent laboratory tests have been cited in the literature. In the evaluation of diagnostic accuracy of WBC and CRP levels, time interval between the onset of the abdominal pain and analysis of these parameters should be taken into consideration.

## 5. Conclusions

Making a diagnosis of appendicitis is a challenging task in the pediatric age group. Since the incidence of perforation is higher in children, timely diagnosis will allow surgical intervention before development of perforation, and also it will be possible to refrain from performing negative appendectomies.

For complicated appendicitis, CRP has the highest degree of diagnostic accuracy.

If WBC and CRP values are within normal limits, even though diagnosis of AA can not be ruled out, diagnosis of complicated appendicitis is a very remote possibility. The diagnosis of appendicitis should be made primarily based on clinical examination, and obviously more specific and systemic inflammatory markers are needed.

Combined use of cut-off values of WBC (≥13100/*μ*L) and CRP (≥1.17 mg/L) yields a higher sensitivity and NPV for the diagnosis of complicated appendicitis.

## Figures and Tables

**Table 1:** General characteristics of the groups.

		Age	Gender	
			Male	Female
		Mean ± SD (median) [min–max]	*n*	*n*
Group 1	126	11.5 ± 2.0 (6–16)	77 (61.1)	49 (38.9)
Group 2	100	10.2 ± 3.3 (6–17)	63 (63.0)	37 (37.0)
Group 3	39	10.3 ± 3.4 (6–16)	22 (56.4)	17 (43.6)
Group 4	199	11.3 ± 3.0 (6–17)	135 (67.8)	64 (32.2)
Group 5	79	11.3 ± 2.9 (6–17)	52 (65.8)	27 (34.2)
*p*		0.010	0.583	

**Table 2:** Laboratory values of the groups in detail.

	WBC × 1000 (4.5–11)		CRP (0–0.6) mg/dL	
	Mean ± SD	95% CI	Mean ± SD	95% CI
Group 1	8.8 ± 2.6	8.3–9.2	0.4 ± 0.7	0.2–0.5
Group 2	11.0 ± 5.4	10.0–12.1	1.5 ± 2.9	0.9–2.1
Group 3	13.3 ± 5.7	11.5–15.1	3.0 ± 4.5	1.5–4.4
Group 4	15.8 ± 4.3	15.2–16.4	4.3 ± 7.2	3.3–5.3
Group 5	16.5 ± 4.5	15.5–17.5	9.7 ± 10.1	7.5–12.0
*p*	<0.001		<0.001	

**Table 3:** Correlation between groups as for WBC and CRP values in combination.

Laboratory markers	Group 1	Group 2	Group 3	Group 4	Group 5	Total
E WBC E CRP	3	22	16	119	63	223
E WBC N CRP	25	19	10	56	8	118
N WBC E CRP	22	13	7	19	7	68
N WBC N CRP	76	46	6	5	1	134
Total	126	100	39	199	79	543

E: elevated; N: normal.
